# Deep brain stimulation for monogenic Parkinson’s disease: a systematic review

**DOI:** 10.1007/s00415-019-09181-8

**Published:** 2019-01-18

**Authors:** Tomi Kuusimäki, Jaana Korpela, Eero Pekkonen, Mika H. Martikainen, Angelo Antonini, Valtteri Kaasinen

**Affiliations:** 1 Division of Clinical Neurosciences, Turku University Hospital, Hämeentie 11, POB 52, 20521 Turku, Finland; 2 Department of Neurology, University of Turku, Turku, Finland; 3 Department of Neurology, Helsinki University Hospital, Helsinki, Finland; 4 Department of Clinical Neurosciences (Neurology), University of Helsinki, Helsinki, Finland; 5 Department of Neurosciences, University of Padua, Padua, Italy

**Keywords:** Parkinson’s disease, Monogenic, Genetic, Deep brain stimulation

## Abstract

Deep brain stimulation (DBS) is an effective treatment for Parkinson’s disease (PD) patients with motor fluctuations and dyskinesias. The key DBS efficacy studies were performed in PD patients with unknown genotypes; however, given the estimated monogenic mutation prevalence of approximately 5–10%, most commonly *LRRK2, PRKN, PINK1* and *SNCA*, and risk-increasing genetic factors such as *GBA*, proper characterization is becoming increasingly relevant. We performed a systematic review of 46 studies that reported DBS effects in 221 genetic PD patients. The results suggest that monogenic PD patients have variable DBS benefit depending on the mutated gene. Outcome appears excellent in patients with the most common *LRRK2* mutation, p.G2019S, and good in patients with *PRKN* mutations but poor in patients with the more rare *LRRK2* p.R1441G mutation. The overall benefit of DBS in *SNCA, GBA* and *LRRK2* p.T2031S mutations may be compromised due to rapid progression of cognitive and neuropsychiatric symptoms. In the presence of other mutations, the motor changes in DBS-treated monogenic PD patients appear comparable to those of the general PD population.

**Electronic supplementary material:**

The online version of this article (10.1007/s00415-019-09181-8) contains supplementary material, which is available to authorized users.

## Introduction

Deep brain stimulation (DBS) provides symptomatic motor benefit for patients with advanced Parkinson’s disease (PD) [1–4]. The benefit of symptom control through DBS surpasses that of optimal medical treatment in patients with motor fluctuations and dyskinesias, and it is a relatively safe treatment option for motor complications of idiopathic PD [1–5]. DBS is often performed in relatively early-onset PD, a population in which it has been estimated that at least 5–10% of cases are not sporadic, but may carry genetic mutations [6, 7]. Genetic cases often are phenotypically different compared to sporadic patients, and this factor may influence clinical outcome [6, 8].

Though DBS has demonstrated efficacy, randomized studies have been performed in PD patients without genetic characterization raising questions of suitability of various monogenic forms and their relevance in DBS outcome. It is known that medication effects may vary between different mutations. For example, patients with *PRKN* mutations generally are particularly prone to levodopa-induced dyskinesias, whereas patients with *LRRK2* mutations tend to show a normal sustained benefit for levodopa [8–11]. The effects of other antiparkinsonian drugs, such as rasagiline, may also be modulated by the genotype [12]. Given the variability in medication effects, it is conceivable that there are also differences in the treatment response to DBS in advanced monogenic PD. There are several case reports and small case series of DBS outcomes in patients with genetic PD, but due to a lack of information synthesis, we performed a systematic review on the effects of DBS in genetic PD.

## Methods

### Search strategy

The Preferred Reporting Items for Systematic Reviews and Meta-Analyses (PRISMA) statement was followed [13]. We performed a PubMed search from inception to June 26, 2018 with keywords “deep brain stimulation or DBS”, “Parkinson’s or Parkinson or Parkinsonism” and “genetic or gene or GBA or PRKN or PARKIN or LRRK2 or SNCA or PINK1 or VPS35 or DJ-1 or UCHL1 or GIGYF2 or HTRA2 or TMEM230 or CHCHD2 or RIC3 or ATP13A2 or PLA2G6 or FBX07 or SYNJ1 or VPS13C or DNAJC6”. All original English language articles concerning genetic PD patients treated with DBS were included. Animal studies and review articles were excluded.

**Fig. 1:**
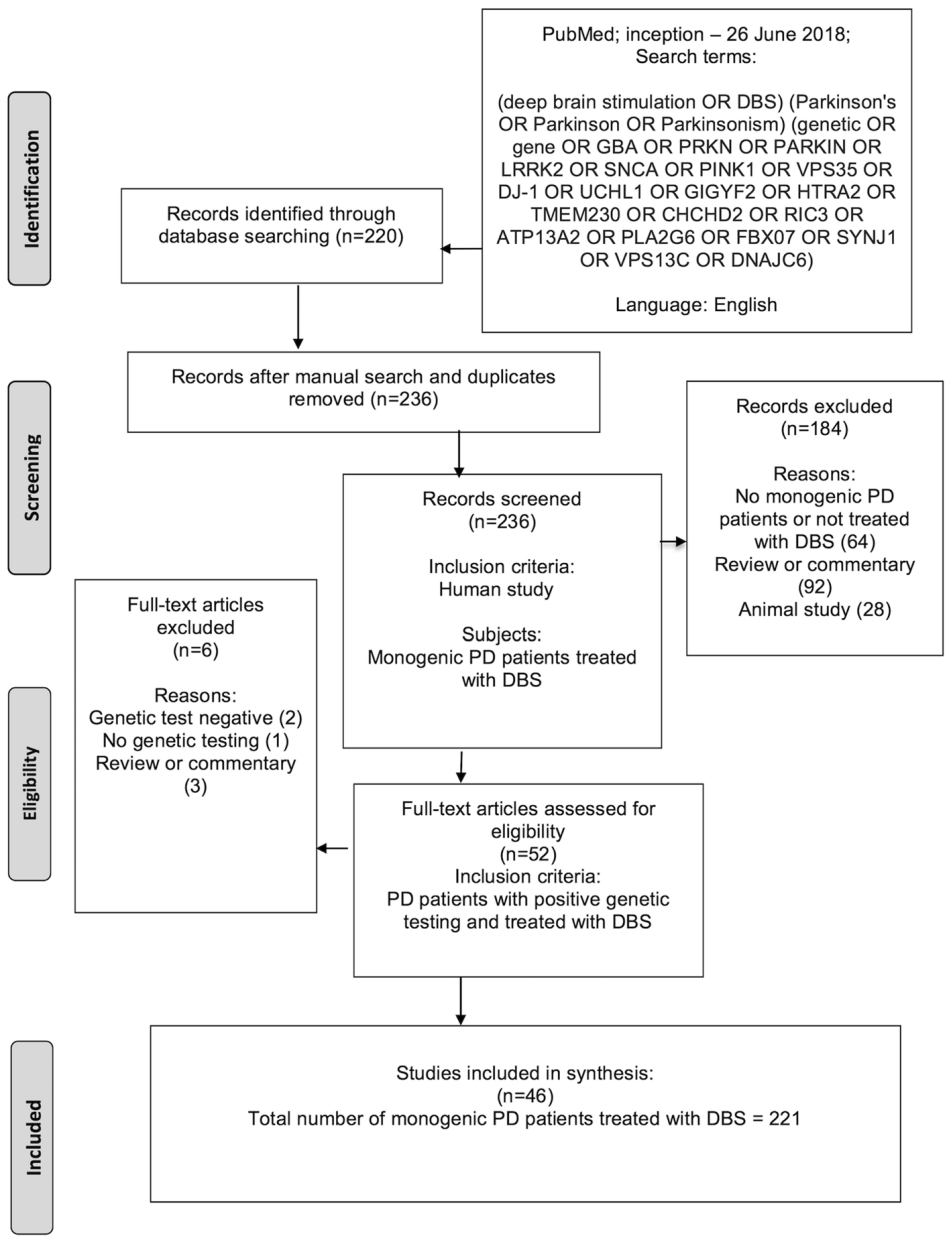
Flow chart of study inclusion and exclusion

The initial search identified 220 articles, and we included an additional 16 relevant studies found in the manual search of reference lists (Fig. 1). All abstracts of these studies were screened, and 184 studies were excluded in the first round (no monogenic PD patients or not treated with DBS *n* = 64, review or commentary article *n* = 92, animal study *n* = 28). The remaining 52 studies were assessed fully for eligibility and six more studies were excluded in the second round (genetic test negative *n* = 2, no genetic testing *n* = 1, review or commentary article *n* = 3). Finally, 46 studies of these 236 studies met all selection criteria and were included in the systematic review (Table 1). A summary of the included studies is presented in Table 2. The included studies reported 221 genetic PD patients who were treated with DBS. However, two studies reported partially the same patients [14, 15].

**Table 1:** The data extracted from the included studies

Study	*N*	Gene	Mutation^a^	AAO^a^	AAD^a^	Target^a^	LP	PRE-UPDRS III^a^	POST-UPDRS III^a^	%^b^	FU	NOS	Outcome
Healy et al. [9]	18	*LRRK2*	p.G2019S	NA	NA^A^	STN^c^	NA	NA	NA	NA	NA	4	Good or excellent (*n* = 8), moderate (*n* = 2), poor (*n* = 2) and NA (*n* = 6)
Sayad et al. [16]	15	*LRRK2*	p.G2019S	40.1 ± 9.4	NA	STN bilat.	+	55.8 ± 16.4 M−, 25.0 ± 13.2 M+ (NC: 51.7 ± 14.4 M−)	27.3 ± 20.6 M−S+, 19.7 ± 18.8 M+S+ (NC: 38.5 ± 16.6 M−S+)	51.1 (NC: 25.5)	2	10	Favourable and better outcome compared to patients without mutation
Greenbaum et al. [17]	13	*LRRK2*	p.G2019S	49.5 ± 6.8	61.1 ± 6.6	STN bilat.	+	42.5 ± 11.8 M−, 19.5 ± 13 M+ (NC: 43.4 ± 12.3 M−)	Short FU 28.5 ± 13.1 M−S+, 17.4 ± 12.9 M+S+ Long FU 30.5 ± 12.8 M−S+, 21.2 ± 9.2 M+S+ (NC: Short FU 27.2 ± 14.1 M−S+, Long FU 33.9 ± 16.1 M−S+)	Short FU 32.8 ± 31.1 Long FU 28.5 ± 32.9 (NC: Short FU 35.6 ± 25.3, Long FU 17 ± 37.1)	0.5–1 (*n* = 13), 3 (*n* = 11)	10	Favourable and comparable to patients without mutations. One patient reported new/worse psychiatric symptoms at 3-year follow-up
Schüpbach et al. [18]	9	*LRRK2*	p.G2019S (*n* = 7) p.G2019S + het. *PRKN* mutation (*n* = 1), p.T2031S (*n* = 1)	33–48	38–65	STN bilat.	NA	41.4 ± 12.4 M−, 8.2 ± 4.6 M+ (NC: 43.4 ± 17.0 M−)	47.7 ± 13.1 M−S−, 17.8 ± 9.6 M−S+, 11.8 ± 4.5 M+S−, 6.2 ± 3.9 M+S+ (NC: 15.7 ± 9.0)	50 ± 36 (NC: 64)	9–10 (Long-term FU for two patients)	10	Favourable and comparable to patients without mutations, but cognitive, behavioral and psychotic problems in the patient with p.T2031S mutation after 5 years
Pal et al. [19]	5	*LRRK2*	NA	47.5 ± 11.0 (*n* = 4)	60.8 ± 9.0 (*n* = 4)	NA	NA	NA	30.8 ± 11.7 M+S+ (*n* = 4)	NA	3.5 ± 2.4 (*n* = 4)	6	The outcome is not reported. Clinical data before DBS is not available, but UPDRS III score was higher in LRRK2 -patients compared to patients without mutations at follow-up
Angeli et al. [15]	5	*LRRK2*	p.G2019S (*n* = 4), p.G2019S + *GBA*-E326K (*n* = 1)	35–55	NA^B^	STN^c^	NA	65.4 ± 14.9 M−, 10.8 ± 5.1 M+ (NC: 47.6 ± 14.8 M−)	69.2 ± 12.4 M−S−, 30.6 ± 16.1 M−S+ (24.6 ± 11.3 M−S+)	53 (NC: 48)	1–5	9	Favourable and comparable to patients without mutations. No reported cognitive problems
Gómez-Esteban et al. [20]	4	*LRRK2*	p.R1441G	29–55	41–65	STN bilat.	+	48.5 ± 18.5 M−, 18.0 ± 7.4 M+ (NC: 42.5 ± 10.6 M−)	39.7 ± 17.7 M−S+, 16.0 ±/–7.7 M+S+ (NC: 26.1 ± 8.4 M−S+)	18 (NC: 39)	0.5	10	Poorer response compared to patients without mutation
Johansen et al. [21]	3	*LRRK2*	p.G2019S	43–57	50–69	STN bilat.	+	NA for individual genes (NC: 35.7 ± 6.7 M−)	NA for individual genes (NC: 19.7 ± 5.5 M−S+)	NA (NC: 44.8)	5	9	Favourable and comparable to patients without mutations
Lesage et al. [22]	3	*LRRK2*	p.G2019S (*n* = 2), p.T2031S (*n* = 1)	34–45	41–66	STN^c^	NA	14 M+ (*n* = 1), NA (*n* = 2)	27 M−S+ (*n* = 1), 17 M−S+ and 32 M−S− (*n* = 1)	NA	7 (Long-term FU for one patient)	9	Favourable to motor symptoms, but depression and psychosis in the patient with p.T2031S mutation
Gaig et al. [23]	3	*LRRK2*	p.G2019S	33–62	NA	STN bilat.	NA	NA	NA	NA	NA	5	Favourable to motor symptoms
Goldwurm et al. [24]	3	*LRRK2*	p.G2019S	NA	NA	NA	NA	NA	NA	NA	NA	2	NA
Hatano et al. [25]	1	*LRRK2*	p.R1441G and p.G2385R	28	39	STN bilat.	+	NA	NA	NA	2	7	Poor motor response with severe psychiatric problems at 1 year after operation
Stefani et al. [26]	1	*LRRK2*	Het. p.G2019S	49	56	STN bilat.	+	27 M−, 12 M+	25 M−S−, 8 M−S+, 5 M+S+	70.4	0.25	8	Favourable outcome
Puschmann et al. [27]	1	*LRRK2*	p.N1437H (c.4309A > C)	50	69	STN bilat.	+	NA	65 M−S+	NA	0.5	8	Poor motor outcome. Patient had also severe depression and suicidality and she finally committed suicide 6.5 months after DBS implantation
Perju-Dumprava et al. [28]	1	*LRRK2*	p.Y1699C	43	48	STN bilat.	NA	54 M−, 32 M+	26 M−S+, 15 M+S+	52 M−, 53 M+	2.5	10	Favourable outcome. No changes in neuropsychological test parameters 6 months postoperatively
Breit et al. [29]	1	*LRRK2*	p.R793M	42	60	STN bilat.	NA	NA	NA	64 (1 year), 56 (8 year)	8	8	Favourable outcome
Aasly et al. [30]	1	*LRRK2*	p.Asn1437His	NA	NA	STN^c^	NA	NA	NA	NA	NA	4	Favourable outcome
Lohmann et al. [31]	14	*PRKN*	One mutation: ex6hetdupl, ex6hetdel, Arg256Cyshet [*n* = 2], Ala398Thrhet, ex7hetdupl, and exhet3del; Hom. or compound het.: ex5hetdel—c.255delAhet, ex3hetdel—prom-ex1hetdel, ex2-4hetdupl—ex3hetdel, Cys289Glyhom, ex5hetdel—Cys441Arghet, ex2hetdel—ex3hetdel and ex4-7hetdel—IVS7-1GC	14–52	32–67	STN bilat.	NA	One mutation 54.3 ± 13.9 M−, 11.6 ± 12.7 M+ Two mutations 55.4 ± 17.3 M−, 14.5 ± 10 M+ (NC: 51.9 ± 18.3 M−)	One mutation 38.4 ± 16.8 M−S− 12.7 ± 11.2 M+S−, 17.8 ± 11.2 M−S+, 10.8 ± 10.1 M + S+ Two mutations 47.7 ± 12.8 M−S−, 17 ± 10.9 M−S+, 14.5 ± 12.5 M−S+, 9.3 ± 8.6 M+S+ (NC: 17.9 ± 15.1 M−S+)	One mutation 69 ± 15 Two mutations 77 ± 14 (MC: 65.5)	1–2 except 3 years for one patient with two PRKN mutations	10	Motor response was favourable and comparable to patients without mutations, but more cognitive problems in homozygous and compound heterozygous patients compared to patients without mutations
Moro et al. [32]	11	*PRKN*	One mutation: delEx6, duplEx5, 867C > T, 1306G > C, delEx5-12; Hom. or compound het.: 202delA [*n* = 2], delEx3-4, delEx3 + 1142-3delGA, delEx2-5 + duplEx8, delEx7-9	15–40	31–66	STN bilat.	NA	35–66 (MV = 49.5)	NA	Short FU 36 Long FU 42 (NC: Short FU 56, Long FU 44)	3–6	9	Favourable and comparable to patients without mutations in long-term follow-up
Pal et al. [19]	10	*PRKN*	NA	30.6 ± 9.1	47.0 ± 11.5	NA	NA	NA	33.8 ± 20.5 M+S+ (*n* = 6)	NA	4.0 ± 4.2	6	The outcome is not reported. Clinical data before DBS is not available but UPDRS III score was higher in PRKN -patients compared to patients without mutations at follow-up
Angeli et al. [15]	5	*PRKN*	Hom.: c.101_102delAG, c.1289G > A p.G430D and c.823C > T, p.Arg275Trp, c.337_376del and c.465–466del, Hom. deletion of exon 3 and 4, c.823C > T; p.Arg275Trp and het. duplication of exon 6	7–36	NA^B^	GPi (*n* = 3), STN^c^ (*n* = 2)	NA	All 57.0 ± 11.2 M−, 21.0 ± 6.4 M+ GPi 53.3 ± 13.9 M− STN 62.5 ± 3.5 M− (NC: STN: 47.6 ± 14.8 M−GPi: 40.5 ± 13.4 M−)	GPi 43.3 ± 16.4 M−S− 42.0 ± 19.0 M−S+ 27.3 ± 17.6 M+S+ STN 84.0 ± 22.6 M-S− 43.0 ± 0.0 M-S+ 23.5 ± 6.4 M+S+ (NC: STN: 24.6 ± 11.3 M-S+, GPi: 51.0 ± 7.1 M-S+)	GPi 21 STN 31 (NC: STN: 48, GPi: − 28)	1–5	9	Good to motor symptoms without cognitive problems. The percentage improvement in the UPDRS III score was better with STN-DBS than with GPi-DBS
Romito et al. [11]	5	*PRKN*	G828A and Dupl ex1, DelAG 202-203, C1101T, G535A, Dupl ex1	27–45	42–63	STN bilat.	+	57.3 ± 9.3 M− 22.8 ± 7.3 M+ (NC: 59.7 ± 11.3 M−)	25.2 ± 10.0 M−S+ 21.8 ± 7.5 M+S+ (NC: 29.0 ± 12.3 M−S+)	56 (NC: 51.4)	1–3	10	Favourable and comparable to patients without mutations
Johansen et al. [21]	4	*PRKN*	Het. c.delEx3, Het. p.R275W, Het. c.duplEx7, Hom. c.delEx5 (GPi)	35–46	50–59	STN bilat. (*n* = 3), GPi unilat. (*n* = 1)	+	NA for individual genes (NC: 35.7 ± 6.7 M−)	NA for individual genes (NC: 19.7 ± 5.5 M−S+)	NA (NC: 44.8)	5–7	9	Favourable and comparable to patients without mutations
Kim et al. [33]	3	*PRKN*	NA	21.7 ± 8.5	49.7 ± 16.2	STN bilat.	NA	49.8 ± 24.5 M−, 18.3 ± 7.8 M+ (NC: 38.3 ± 10.6 M−)	24.7 ± 14.0 M−S+, 22.2 ± 14.9 M+S+ (NC: 17.2 ± 5.5 M−S+)	37.1 ± 45.4 (NC: 54.6 ± 13.9)	2–5	10	Favourable and comparable to patients without mutations
Hassin-Baer et al. [34]	3	*PRKN*	Hom. 202 A deletion	15–28	31–54	STN^c^	NA	27–64 M−, 20–48 M+	NA	NA	NA	7	Modest outcome with improvement in appendicular symptoms, but no change in axial features
Sayad et al. [16]	2	*PRKN*	Het. c. 458C > G	48	NA	STN bilat.	+	46 M−, 28 M+	51 M−S+, 30 M+S+	− 10.1	2	10	Poor response
			Het. c. 1204C > T	48			+	49 M−, 32 M+ (NC: 51.7 ± 14.4 M−)	51 M−S+, 47 M+S+ (NC: 38.5 ± 16.6 M−S+)	− 4.1 (NC: 25.5)			
Thompson et al. [35]	2	*PRKN*	Hom., specific mutation NA	26 (Gpi), 30 (STN)	NA	STN bilat. (*n* = 1), GPi bilat. (*n* = 1)	NA	GPi 57 M−, 50 M+ STN 47 M−, 21 M+	NA	NA	3 (STN), 8 (GPi)	6	Favourable outcome
Genç et al. [36]	1	*PRKN*	Het. c89G > A and large het. deletion	10	NA	STN bilat.	+	48 M−, 7 M+	7 M−S+, 4 M+S+	85.4	NA	6	Favourable to motor symptoms
Moll et al. [37]	1	*PRKN*	Compound het. *PRKN* mutation (delExon1 + c.924C > T)	35	45	STN bilat.	+	30 M− 5 M+	NA	NA	NA	7	Favourable to motor symptoms
Nakahara et al. [38]	1	*PRKN* + *PINK1*	Hom. parkin mutation (p.T175PfsX2) + het. PINK1 mutation (p.R58-V59insGR)	15	60	STN bilat.	+	86 M−, 25 M+	33 M−S+, 21 M+S+	62	0.7	9	Favourable outcome
Lefaucheur et al. [39]	1	*PRKN*	Compound het. mutations of the *PRKN* gene, [c.101_102delAG (p.Gln34ArgfsX5) + c.155delA (p.Asn52MetfsX29)]	25	69	STN^c^	NA	NA	NA	55	0.5	8	Favourable to motor symptoms without cognitive problems
Wickremaratchi et al. [40]	1	*PRKN*	Compound het. exon 2/exon 2 1 3 deletion in the *PRKN*	8	46	Zona incerta bilat.	NA	68 M−, 22 M+	NA M−S+ 24 M+S+	NA M−, 64.7 M+	0.5	9	Favourable outcome
Lesage et al. [41]	1	*PRKN*	Compound het. of the *PRKN* c.1-?_7+?del and c.172-?_412+?del mutations	8	39	STN bilat.	NA	46 M−, 15.5 M+	NA	NA	NA	6	Favourable outcome
Capecci et al. [42]	1	*PRKN*	Hom. deletion in exon 3	22	NA	STN bilat.	+	45 M−, 5 M+	7 M−S+, 3 M+S+	84.4	1	8	Favourable outcome
Khan et al. [43]	1	*PRKN*	Exon 9 1101C–>T (Arg334Cys), exon 7 939G–>A (Asp230Asn)	30	35	STN bilat.	NA	NA	NA	NA	NA	6	Favourable outcome
Lythe et al. [14]	17^d^	*GBA*	Het. mutation carriers (*n* = 15), hom. mutation carrier (*n* = 1), compound het. (*n* = 1). Two patients also carried a mutation in another PD-associated gene; *PARKIN* or *LRRK2*	41.4 ± 5.8	53.5 ± 4.5	STN^c^ (*n* = 15), GPi (*n* = 2)	NA	52.4 ± 13.0 M−, 18.4 ± 14.9 M+ (NC: 40.5 ± 12.0 M−)	NA M−S+, 50.0 ± 17.1 M+S+ (*n* = 9) (NC: NA M−S+, 38.9 ± 14.0 M+S+)	4.6 M+S+ (*n* = 9) (NC: 4.0 M+S+)	7.5 (*n* = 9)	9	Follow-up data available for 9 patients. Poorer outcome compared to patients without mutations. GBA mutation carriers had faster rate of cognitive decline, reported significantly worse quality of life and exhibited a greater burden of non-motor symptoms compared to patients without mutations. During follow-up 3 GBA + patients were deceased, 2 were unable to complete follow-up due to severe PD-related disability, 2 could not be contacted and 1 DBS hardware was removed
Angeli et al. [15]	16	*GBA*	R463C/R463C, L444P/E326K, N370S, D409H, recNcil, R463C, N188S, R275Q, IVS2 + 1 G > A, L444P, E326K/E326K, E326K (*n* = 3), E326K and *LRRK2* p.G2019S, T369M and *PRKN* c.1310C > T	34–58	NA^B^	STN^c^ (*n* = 13), GPi (*n* = 2), VIM (*n* = 1)	NA	All 51.3 ± 14.0 M−, 18.0 ± 15.4M+ GPi 64.5 ± 21.9M− STN 50.5 ± 12.4 M− VIM 35 M− (NC: STN: 47.6 ± 14.8 M−GPi: 40.5 ± 13.4 M−)	GPi 66.5 ± 19.1 M−S−, 50.0 ± 19.8 M−S+, 41.0 ± 15.6 M+S+ STN 56.1 ± 18.8 M−S−, 28 ± 11.4 M−S+, 15.9 ± 10.4 M+S+ VIM 35 M−S−, 20 M−S+, 8 M+S+ (NC: STN: 24.6 ± 11.3 M−S+ GPi: 51.0 ± 7.1 M−S+)	GPi 22 STN 40 VIM 43 (NC: STN: 48, GPi: − 28)	1–5	9	Favourable motor response, but faster rate of cognitive decline compared to patients without mutations. The percentage improvement in the UPDRS III score “OFF- medication” was better with bilateral STN-DBS and VIM-DBS than with GPi-DBS
Pal et al. [19]	12	*GBA*	p.N370S (*n* = 8), p.L444P (*n* = 3). 1 patient carried both GBA and LRRK2 mutations and was excluded	41.6 ± 5.3 (*n* = 11)	53.9 ± 2.6 (*n* = 9)	NA	NA	NA	27.4 ± 14.5 M+S+ (*n* = 11)	NA	1.6 ± 3.0 (*n* = 9)	6	The outcome is not reported. Clinical data before DBS is not available, but UPDRS-III score was little higher in GBA -patients compared to patients without mutations at follow-up
Weiss et al. [44]	3	*GBA*	p.N370S (*n* = 1) and p.L444P (*n* = 2)	47–54	65–69	STN^c^	NA	26 and 53 M−, 14 and 19 M+, NA (*n* = 1) (NC: 31–63 M−)	56–71 M−S−, 21–45 M−S+, 32–48 M+S−, 20–45 M+S+ (NC: 21–42 M−S+)	30–75 (NC: 22–54)	6–10	11	Favourable outcome, but substantial increase of axial motor impairment in the long-term with declining therapeutic response in *GBA* carriers. *GBA* carriers developed also a significant cognitive impairment
Lesage et al. [45]	2	*GBA*	Hom. p.N370S	52	NA	STN bilat.	NA	NA	NA	NA	NA	5	Favourable outcome
			c.1263del + RecTL	21	24						2		Some clinical benefit 2 years after DBS, but problems with postural instability
Martikainen et al. [46]	1	*SNCA*	Het. c.158C > A (p.A53E)	42	46	STN bilat.	NA	31 M−, 8 M+	NA	NA	3.5	9	Favourable motor outcome in the short-term but poor in the long-term follow-up. Response for motor fluctuations remained satisfactory but the cognitive and mental state of the patient deteriorated to a state of practical immobility
Perandones et al. [47]	1	*SNCA*	*SNCA* duplication	18	26	GPi bilat.	+	NA	NA	NA	0.1	6	Favourable and comparable to patients without mutations
Shimo et al. [48]	1	*SNCA*	*SNCA* duplication	35	41	STN bilat.	+	27 M−, 10 M+	13 M−S+	51.9	4	9	Favourable motor outcome without cognitive or psychiatric problems
Antonini et al. [49]	1	*SNCA*	*SNCA* duplication at 4q22.1	41	46	STN bilat.	+	28 M−, 10 M+	16 M−S+, 10 M+S+	42.9	2	9	Favourable outcome in short-term follow-up but patient developed visual hallucinations and cognitive deterioration and died two years after operation due to metastatic breast cancer
Ahn et al. [50]	1	*SNCA*	*SNCA* duplication	40	46	STN bilat.	NA	32 M−, 6 M+	NA	NA	NA	6	Excellent motor response but later patient’s dementia worsened, requiring assistance in daily activities
Fleury et al. [51]	2	*VPS35*	p.D620N	49	60	STN bilat.	NA	58 M−, 17 M+	32 M−S−, 18 M−S+, 18 M+S−, 15 M+S+	76 (1 year) 69 (8 years)	8	8	Favourable outcome
				45	55			28 M−, 15 M+	NA	36 (1 year)	1		Tremor, akinesia and rigidity improved markedly but patient’s walking difficulties worsened with an increased frequency of freezing episodes and falls after surgery (problems disappeared after levodopa intake with the STN-DBS switched on)
Chen et al. [52]	1	*VPS35*	p.D620N	42	55	STN bilat.	+	42 M−, 15 M+	35 M−S−, 22 M−S+, 15 M+S−, 13 M+S+	37	5	9	Favourable outcome
Kumar et al. [53]	1	*VPS35*	p.D620N	NA	NA	NA	NA	NA	NA	NA	NA	3	Little benefit to motor symptoms, but patient developed significant dysarthria
Sheerin et al. [54]	1	*VPS35*	p.D620N	47	NA	NA	NA	NA	NA	NA	NA	5	Favourable outcome. No reported cognitive problems
Borellini et al. [55]	1	*PINK1*	Hom. L347P	30	49	GPi	NA	44 M−	32 M + S+	27	0.1	7	Moderate outcome
Nakahara et al. [38]	1	*PRKN* + *PINK1*	Hom. parkin mutation (p.T175PfsX2) + het. PINK1 mutation (p.R58-V59insGR)	15	60	STN bilat.	+	86 M−, 25 M+	33 M−S+, 21 M + S+	62	0.7	9	Favourable outcome
Johansen et al. [21]	1	*PINK1*	Het. p.G411S	50	59	STN bilat.	+	NA for individual genes (NC: 35.7 ± 6.7 M−)	NA for individual genes (NC: 19.7 ± 5.5 M−S+)	NA (NC: 44.8)	5	9	Favourable and comparable to patients without mutations
Moro et al. [32]	1	*PINK1*	Hom. c.509T > G (p.V170G)	31	61	STN bilat.	NA	35.5 M−	NA	Short FU 46.5 Long FU 43.7 (NC: Short FU 56, Long FU 44)	3–6	9	Favourable and comparable to patients without mutations
Valente et al. [56]	1	*PINK1*	NA	NA	NA	STN bilat.	NA	NA	NA	NA	NA	3	Motor outcome was not properly reported but patient developed imbalance, gait impairment, dysarthria, and behavioral changes at the age of 54 years. Mental deterioration was documented a few years later
Dufournet et al. [57]	3		22q11.2 Del. Syndrome	34–38^e^	NA	STN^c^ (*n* = 1) GPi (*n* = 2)	NA	NA	NA	30–70	NA	7	Favourable and comparable to patients with idiopathic PD

*AAO* age at disease onset (years), *AAD* age at DBS operation (years), *LP* specific lead position (reported or not), *%* the percentage improvement of the UPDRS III score after DBS^b^, *FU* follow-up after surgery (years), *NA* not available, *M−/+* medication OFF/ON, *S−/+* stimulation OFF/ON, *MV* mean value, *NC* mutation non-carriers

^A^The mean time from PD onset to surgery was 11.4 years (SD 6.2), ^B^mean duration of PD (years) at DBS assessment: PRKN = 25.2 ± 12.8, GBA = 11.2 ± 5.0, LRRK2 = 12.1 ± 1.8

^a^Parameters are reported in the table as in the original articles

^b^If the percentage improvement was not reported directly in the original article but UPDRS-III scores were available, we calculated the percentage improvement from the change of UPDRS-III score in the preoperative M− condition compared to the postoperative M−S+ condition (((Pre-op. UPDRS-III M−) − (Post.op. UPDRS-III M−S+))/(Pre-op. UPDRS-III M−) × 100)

^c^The study did not specify whether the implantation was uni- or bilateral

^d^Some patients were reported previously by Angeli et al. [15]

^e^Age at PD diagnosis

**Table 2:** Summary of key findings according to the mutated gene

Gene	Studies (*n*)	Patients (*n*)	Target	Outcome
*LRRK2*	17	87^a^	STN: *n* = 79 (90.8%) NA: *n* = 8 (9.2%)	Mostly favourable motor outcome. Four studies with eight patients (9.2%) reported poor motor outcomes and one study reported moderate outcomes for two patients. Both patients with the *LRRK2* p.T2031S (c.6091A > T) mutation (*n* = 2) developed neuropsychiatric problems 5–7 years after implantation. The outcome appears poor in patients with *LRRK2* p.R1441G (c.4321C > G) mutations (*n* = 5), whereas it appears excellent in patients with *LRRK2* p.G2019S (c.6055G > A) mutations
*PRKN*	18	67^b^	STN: *n* = 51 (76.1%) GPi: *n* = 5 (7.5%) Zona incerta: *n* = 1 (1.5%) NA: *n* = 10 (14.9%)	Fifty-one patients (76.1%) had favourable long-term motor outcomes. Four patients (6.0%) were reported to have modest outcome in two different studies and one study with two patients (3.0%) reported poor benefit
*GBA*	5	50^c^	STN: *n* = 33 (66.0%) GPi: *n* = 4 (8.0%) VIM: *n* = 1 (2.0%) NA: *n* = 12 (24.0%)	Eighteen patients were reported to have favourable, three patients moderate and 9 patients poor long-term motor outcomes. One study reported better outcomes with STN-DBS and VIM-DBS than with GPi-DBS. *GBA* mutation carriers developed cognitive impairment faster than patients without mutations
*SNCA*	5	5	STN: *n* = 4 (80.0%) GPi: *n* = 1 (20.0%)	Favourable motor outcome but three of five patients developed cognitive or neuropsychiatric problems a few years after implantation
*VPS35*	4	5	STN: *n* = 3 (60.0%) NA: *n* = 2 (40.0%)	Favourable motor outcome in four cases and minor motor benefit complicated by dysarthria in one case
*PINK1*	5	5^b^	STN: *n* = 4 (80.0%) GPi: *n* = 1 (20.0%)	Favourable motor outcome in three cases and moderate in one case
*22q11.2.Del. Syndrome*	1	3	STN: *n* = 1 (33.3%) GPi: *n* = 2 (66.6%)	Favourable motor outcome

*STN* subthalamic nucleus, *GPi* globus pallidus interna, *VIM* ventral intermediate nucleus, *NA* not available

^a^One patient had also PRKN mutation and one had GBA mutation

^b^One patient had both PRKN and PINK1 mutations

^c^Two studies reported partially same patients, but it was not possible to separate individual patients that were reported twice. One patient had also LRRK2 mutation and one had PRKN mutation

### Specific aims

This review of evidence aimed to systematically investigate DBS outcome in monogenic PD compared to the general PD population. The primary aim was to evaluate the motor benefit of the DBS operation in each monogenic PD type. An additional aim was to evaluate effects on non-motor symptoms, including possible cognitive and neuropsychiatric symptoms.

### Selection criteria

Search terms and the PubMed search were planned by two authors (T.K. and V.K.). All titles and abstracts were reviewed by one investigator (T.K.). Studies were excluded if the title and/or abstract were not suitable for the aim of the review. Full texts were obtained for appropriate studies or if the relevance of an article was uncertain. The inclusion criteria for the selected studies were as follows: (1) a human study, (2) genetic PD patients treated with DBS, and (3) English language. The data extracted from each study were study year, first author’s family name, number of patients, mutated gene, specific mutation, patient age at disease onset and DBS implantation, target nucleus of DBS, more specific lead positioning, pre- and postoperative UPDRS-III scores, follow-up time and outcome (Table 1). UPDRS-III scores of control cohort's (mutation non-carriers, NC) are also reported in Table 1 if the information was available. In the outcome evaluation, an improvement of 30% or more in the UPDRS-III motor score was considered to indicate favourable outcome; 20–30%, moderate outcome; and < 20%, poor/mild outcome [58–60].

### Quality control

The quality of the included studies was evaluated according to the Newcastle-Ottawa Scale (NOS) [61]. NOS includes selection, comparability, and exposure or outcome. The scale ranged from 0 to 11 stars, with the highest rating representing the greatest quality. Six months or more was a limit for the adequate follow-up time. Pre- and postoperative evaluation was thought to be accomplished if the outcome was reported properly with percentage improvement of the UPDRS-III score or verbally. A total score of 0–3 was considered to indicate to poor quality; 4–7, moderate quality; and 8–11, good quality. The NOS total score is presented in Table 1 and the scale is presented more accurately in Supplementary Table 1. A summary of the assessed quality of the studies is presented in Supplementary Table 2.

## Results

A summary of the primary results is presented in Table 2. Altogether, 46 studies and 221 monogenic PD patients treated with DBS were included in the systematic review (Table 1).

### LRRK2

Seventeen studies [9, 15–30] reported 87 patients (target: subthalamic nucleus (STN) *n* = 79, not available (NA) *n* = 8). The outcome was reported in 73 patients (83.9% of patients); with percentage improvement of the UPDRS-III score in 49 patients and verbally in 24 patients. The motor outcome was mostly favourable in patients with *LRRK2* mutation. Only five studies with ten patients reported poor/mild/moderate outcomes. Both patients with the p.T2031S (c.6091A > T) mutation (*n* = 2) developed neuropsychiatric problems 5–7 years after implantation. The outcome appeared poor in patients with p.R1441G (c.4321C > G) mutations whereas it appeared excellent in patients with p.G2019S (c.6055G > A) mutations.

### PRKN

Eighteen studies [11, 15, 16, 19, 21, 31–43] reported 67 patients (STN *n* = 51, globus pallidus interna (GPi) *n* = 5, zona incerta *n* = 1, NA *n* = 10). The outcome was reported in 57 patients (85.1%); UPDRS-III percentage improvement was reported in 45 patients and the outcome was described verbally in 12 patients. Fifty-one patients (76.1%) had favourable long-term motor outcomes. Six patients in three different studies were reported to have modest or poor outcomes.

### GBA

Five studies [14, 15, 19, 44, 45] reported 50 patients (STN *n* = 33, GPi *n* = 4, ventral intermediate nucleus (VIM) *n* = 1, NA *n* = 12). Samples partially consisted of same patients in two studies [14, 15]. The outcome was reported in 30 patients (60.0%); UPDRS-III percentage improvement in 28 patients and the outcome was described verbally in 2 patients. Eighteen patients were reported to have favourable, three patients moderate and nine patients poor long-term motor outcomes. One study reported better outcomes with STN-DBS and VIM-DBS than with GPi-DBS [15]. GBA mutation carriers developed cognitive impairment faster than patients without mutations.

### SNCA

Five patients were reported in five case reports [46–50] (STN *n* = 4, GPi *n* = 1). The motor outcome was favourable for all patients in the short-term but 3/5 patients developed cognitive and/or neuropsychiatric problems a few years after implantation. The percentage change in the UPDRS-III score was documented in two patients.

### VPS35

Four studies [51–54] reported five patients (STN *n* = 3, NA *n* = 2). Favourable motor outcome was reported in four cases and minor motor benefit complicated by dysarthria in one case. The percentage change in the UPDRS-III score was reported in three patients.

### PINK1

Five case reports [21, 32, 38, 55, 56] including one patient in each report (STN *n* = 4, GPi *n* = 1) were reported. Favourable motor outcome was observed in three patients and moderate outcome in one case. One patient developed imbalance, gait impairment, dysarthria, and behavioral changes after operation and mental deterioration was documented a few years later.

### Exclusion of poorer quality studies

Unfortunately, many studies (Table 1) lacked important information as shown in the Supplementary Table 1. Poorer quality studies have tendency for bias; therefore, in the Supplementary Table 3, data are presented after exclusion of poorer quality studies such as studies lacking the information about DBS target, pre- and postoperative evaluation, adequate follow-up time or outcome information. Furthermore, as Lythe et al. [14] and Angeli et al. [15] reported partly the same patients, we tested the conclusions also when the smaller study was excluded. Nevertheless, after the exclusion of these studies, the results remained essentially the same (Supplementary Table 4).

## Discussion

We report the following key findings: (1) DBS outcome appears excellent in patients with *LRRK2* p.G2019S (c.6055G > A) mutations, good in patients with *PRKN* mutations and poor in patients with *LRRK2* p.R1441G (c.4321C > G) mutations, (2) the overall benefit of DBS in *SNCA, GBA* and *LRRK2* p.T2031S (c.6091A > T) mutations may be decreased due to rapid progression of cognitive and neuropsychiatric symptoms, and (3) in other mutations, the motor outcome in DBS-treated genetic PD patients appears generally comparable to that of sporadic PD patients.

A recent smaller review of 30 studies described the effects of DBS mainly in patients with *LRRK2, PRKN* and *GBA* mutations [62]. In the present PRISMA-compliant systematic review of 46 studies and 221 patients, the most comprehensive data were available for patients with *LRRK2* and *PRKN* mutations. The combined evidence suggests that patients with *LRRK2* mutations generally have a good response to DBS, and patients with the most common *LRRK2* mutation, the p.G2019S mutation [7], may even have better outcome than the general PD population. However, the reported *LRRK2* cases of p.R114G, p.T2031S and p.N1437H (c.4309A > C) mutation carriers appeared to have less favourable outcome. This interpretation is limited by the small number of reported DBS-treated cases of rarer *LRRK2* mutations. For the *PRKN* mutations, the literature supports a view that patients with *PRKN* mutations are optimal candidates for DBS.

Apart from the *LRRK2* and *PRKN* genes, the published literature concerning individual monogenic mutations and DBS is less comprehensive and the data are clearly limited with respect to both the number of patients and duration of follow-up. The available data are limited to five DBS-treated patients with *VPS35* mutation, and the patients have shown favourable sustained motor outcome in 4/5 cases. The available literature also suggests that most patients with mutations in *GBA* tend to achieve favourable long-term motor outcome from STN-DBS. Despite good motor outcome, *GBA* mutation carriers may develop cognitive impairment after DBS faster than patients without mutations. *SNCA* patients commonly develop cognitive and neuropsychiatric problems [8]. The literature supported a good motor outcome after DBS also in patients with SNCA mutations; however, 3/5 patients developed cognitive and neuropsychiatric problems a few years after DBS implantation. Indeed, the non-motor features of genetic PD may be a limiting factor in the overall benefit of DBS in some mutations, such as *SNCA* and *LRRK2* p.T2031S. While the motor benefit from DBS may initially be clear, the rapid non-motor progression may lessen the sum value for the quality of life. A recent study in *SNCA* A53T mutated rodents suggested that DBS may be neuroprotective [63]. Nonetheless, in human PD patients with *SNCA* mutations, the neuropsychiatric progression appears to be rapid despite DBS. The issue could be the level of damage at the time of implantation, and earlier DBS in these patients might possibly provide different outcomes.

Preoperative response to levodopa is the best single predictor of the postoperative outcome of DBS [64]. This indicator appears useful also in patients with monogenic mutations and the response was reported in practically all included studies. Another relevant predictor is the localization of DBS electrodes [65]. Unfortunately, there were studies, which did not report DBS targets and most studies lacked information about lead positioning. As the literature expands in the future, the effect of targets and lead positioning should be investigated in more detail. In most studies, STN was preferred over GPi as the target. Hence it remains ambiguous whether there are any relevant differences of clinical outcome between STN and GPi stimulation in monogenic PD. One study reported also a patient with VIM stimulation which is an unusual target for PD patients because VIM stimulation improves only tremor, not other PD symptoms [66, 67]. Finally, it is important to note that the genetic status may have a positive as well as a negative influence on outcome of surgery and this issue should be taken into consideration in the interpretation of DBS studies. For example, the EARLYSTIM trial was performed with young-onset PD patients [5] and there could have been an overrepresentation of PRKN patients in the sample.

In conclusion, monogenic PD patients have variable DBS outcomes depending on the mutated gene. Most patients benefit from STN-DBS, at least in the short-term; however, the current evidence does not support or is questionable for DBS implantation for patients with p.T2031S or p.R114G mutations in the *LRRK2* gene or mutations in the *SNCA* or *GBA* genes. The best outcome from DBS surgery appears to be in patients with *LRRK2* p.G2019S or *PRKN* mutations.

## Electronic supplementary material

Below is the link to the electronic supplementary material.

Supplementary material 1 (DOCX 131 KB)
