# Supplementary material for: Deep brain stimulation for monogenic Parkinson’s disease: a systematic review
Source: J Neurol. 2019 Jan 18;267(4):883–97. doi: 10.1007/s00415-019-09181-8 (PMC7109183; doi:10.1007/s00415-019-09181-8)
Supplement: Supplementary file 1 — Supplementary material 1 (DOCX 131 KB) [file 415_2019_9181_MOESM1_ESM.docx]

**Supplementary material**

**Journal of Neurology**

**Deep brain stimulation for monogenic Parkinson’s disease: A systematic review**

Tomi Kuusimäki^1,2,*^ MD, Jaana Korpela^1,2^ MD, PhD, Eero Pekkonen^3^ MD, PhD, Mika H. Martikainen^1,2^ MD, PhD, Angelo Antonini^4^ MD, PhD, and Valtteri Kaasinen^1,2^ MD, PhD

1. Division of Clinical Neurosciences, Turku University Hospital, Turku, Finland
2. Department of Neurology, University of Turku, Turku, Finland
3. Department of Neurology, Helsinki University Hospital and Department of Clinical Neurosciences (Neurology), University of Helsinki, Helsinki, Finland
4. Department of Neurosciences, University of Padua, Padua Italy

* Corresponding author: Tomi Kuusimäki, Division of Clinical Neurosciences, Turku University Hospital, Hämeentie 11, POB 52, FIN-20521, Turku, Finland, +358-2-3130000, Email: tomi.kuusimaki@utu.fi.

ORCID: 0000-0002-4829-7275

**Supplementary Table 1.** Assessed quality of the included studies.

| Study | Gene | Specific mutation | AAO | AAD | Target | UPDRS score | Non-motor symptoms | Pre- and postoperative evaluation | Tests under blind randomized conditions | Cases and controls matched | Adequate follow-up time | Total score |
| --- | --- | --- | --- | --- | --- | --- | --- | --- | --- | --- | --- | --- |
| Healy et al. 2008[1] | * | * |  |  | * |  |  | * |  |  |  | 4 |
| Sayad et al. 2016 [2] | * | * | * |  | * | * | * | * | * | * | * | 10 |
| Greenbaum et al. 2013 [3] | * | * | * | * | * | * | * | * |  | * | * | 10 |
| Schüpbach et al. 2007 [4] | * | * | * | * | * | * | * | * |  | * | * | 10 |
| Pal et al.  2016 [5] | * |  | * | * |  | * | * |  |  |  | * | 6 |
| Angeli et al. 2013 [6] | * | * | * |  | * | * | * | * |  | * | * | 9 |
| Gómez-Esteban et al. 2008 [7] | * | * | * | * | * | * | * | * |  | * | * | 10 |
| Johansen et al. 2011 [8] | * | * | * | * | * |  | * | * |  | * | * | 9 |
| Lesage et al. 2007 [9] | * | * | * | * | * | * | * | * |  |  | * | 9 |
| Gaig et al. 2006 [10] | * | * | * |  | * |  |  | * |  |  |  | 5 |
| Goldwurm et al. 2005 [11] | * | * |  |  |  |  |  |  |  |  |  | 2 |
| Hatano et al. 2014 [12] | * | * | * | * | * |  | * |  |  | X | * | 7 |
| Stefani et al. 2013 [13] | * | * | * | * | * | * | * | * |  | X |  | 8 |
| Puschmann et al. 2012 [14] | * | * | * | * | * |  | * | * |  | X | * | 8 |
| Perju-Dumprava et al. 2012 [15] | * | * | * | * | * | * | * | * |  | X | * | 10 |
| Breit et al. 2010 [16] | * | * | * | * | * | * |  | * |  | X | * | 8 |
| Aasly et al. 2010 [17] | * | * |  |  | * |  |  | * |  |  |  | 4 |
| Lohmann et al. 2008 [18] | * | * | * | * | * | * | * | * |  | * | * | 10 |
| Moro et al. 2008 [19] | * | * | * | * | * | * |  | * |  | * | * | 9 |
| Romito et al. 2005 [20] | * | * | * | * | * | * | * | * |  | * | * | 10 |
| Kim et al. 2014 [21] | * |  | * | * | * | * | * | * | * | * | * | 10 |
| Hassin-Baer et al. 2011 [22] | * | * | * | * | * |  | * | * |  |  |  | 7 |
| Thompson et al. 2013 [23] | * |  | * |  | * |  | * | * |  |  | * | 6 |
| Genç et al. 2016 [24] | * | * | * |  | * | * |  | * |  | X |  | 6 |
| Moll et al. 2015 [25] | * | * | * | * | * |  | * | * |  | X |  | 7 |
| Nakahara et al. 2014 [26] | * | * | * | * | * | * | * | * |  | X | * | 9 |
| Lefaucheur et al. 2010 [27] | * | * | * | * | * |  | * | * |  | X | * | 8 |
| Wickremaratchi et al. 2009 [28] | * | * | * | * | * | * | * | * |  | X | * | 9 |
| Lesage et al. 2007 [29] | * | * | * | * | * |  |  | * |  |  |  | 6 |
| Capecci et al. 2004 [30] | * | * | * |  | * | * | * | * |  | X | * | 8 |
| Khan et al. 2003 [31] | * | * | * | * | * |  |  | * |  |  |  | 6 |
| Lythe et al. 2017 [32] | * |  | * | * | * | * | * | * |  | * | * | 9 |
| Weiss et al. 2012 [33] | * | * | * | * | * | * | * | * | * | * | * | 11 |
| Lesage et al. 2011 [34] | * | * | * |  | * | * |  |  |  |  |  | 5 |
| Martikainen et al. 2015 [35] | * | * | * | * | * | * | * | * |  | X | * | 9 |
| Perandones et al 2015 [36] | * | * | * | * | * |  |  | * |  | X |  | 6 |
| Shimo et al. 2014 [37] | * | * | * | * | * | * | * | * |  | X | * | 9 |
| Antonini et al. 2012 [38] | * | * | * | * | * | * | * | * |  | X | * | 9 |
| Ahn et al. 2008 [39] | * | * | * | * | * |  | * |  |  | X |  | 6 |
| Fleury et al. 2013 [40] | * | * | * | * | * | * |  | * |  | X | * | 8 |
| Chen et al. 2017 [41] | * | * | * | * | * | * | * | * |  | X | * | 9 |
| Kumar et al. 2012 [42] | * | * |  |  |  |  |  | * |  | X |  | 3 |
| Sheerin et al. 2012 [43] | * | * | * |  |  |  | * | * |  | X |  | 5 |
| Borellini et al. 2017 [44] | * | * | * | * | * | * |  | * |  | X |  | 7 |
| Valente et al. 2004 [45] | * |  |  |  | * |  |  | * |  |  |  | 3 |
| Dufournet et al. 2017 [46] | * | * | * |  | * | * | * | * |  |  |  | 7 |

^The scale ranged from zero to eleven stars, with the highest rating representing the greatest quality. A total score of 0-3 was considered to indicate poor quality; 4-7, moderate quality; and 8-11, good quality. AAO = Age at onset of the disease, AAD = Age at DBS operation, X = Case report^

| *Gene* | *Studies (n)* | *0-3 stars* | *4-7 stars* | *8-11 stars* |
| --- | --- | --- | --- | --- |
| *LRRK2* | 17 | 1 | 5 | 11 |
| *PRKN* | 18 | 0 | 7 | 11 |
| *GBA* | 5 | 0 | 2 | 3 |
| *SNCA* | 5 | 0 | 2 | 3 |
| *VPS35* | 4 | 1 | 1 | 2 |
| *PINK1* | 5 | 1 | 1 | 3 |
| *22q11.2 Del Syndrome* | 1 | 0 | 1 | 0 |

^The scale ranged from zero to eleven stars, with the highest rating representing the greatest quality. A total score of 0-3 was considered to indicate poor quality; 4-7, moderate quality; and 8-11, good quality.^

| *Study* | *N* | *Gene* | *Mutation** | *AAO** | *AAD** | *Target** | *LP* | *PRE-UPDRS III** | *POST-UPDRS III** | *%*** | *FU* | *NOS* | *Outcome* |
| --- | --- | --- | --- | --- | --- | --- | --- | --- | --- | --- | --- | --- | --- |
| Sayad et al. 2016 [2] | 15 | *LRRK2* | p.G2019S | 40.1 ± 9.4 | NA | STN bilat. | + | 55.8 ± 16.4 M-, 25.0 ± 13.2 M+  (NC: 51.7 ± 14.4 M-) | 27.3 ± 20.6  M-S+, 19.7 ± 18.8 M+S+  (NC: 38.5 ± 16.6 M-S+) | 51.1  (NC: 25.5) | 2 | 10 | Favourable and better outcome compared to patients without mutation. |
| Greenbaum et al. 2013 [3] | 13 | *LRRK2* | p.G2019S | 49.5 ± 6.8 | 61.1 ± 6.6 | STN bilat. | + | 42.5 ± 11.8 M-, 19.5 ± 13 M+  (NC: 43.4 ± 12.3 M-) | *Short FU:* 28.5 ± 13.1  M-S+, 17.4 ± 12.9 M+S+  *Long FU:*  30.5 ± 12.8  M-S+, 21.2 ± 9.2 M+S+  (NC:  Short FU 27.2 ± 14.1 M-S+  Long FU 33.9 ± 16.1 M-S+) | *Short FU:*  32.8 ± 31.1  *Long FU:*  28.5 ± 32.9  (NC:  Short FU 35.6 ± 25.3  Long FU 17 ± 37.1) | 0.5 – 1 (n=13), 3 (n=11) | 10 | Favourable and comparable to patients without mutations. One patient reported new/worse psychiatric symptoms at three-year follow-up. |
| Schüpbach et al. 2007 [4] | 9 | *LRRK2* | p.G2019S (n=7),  p.G2019S + het. *PRKN* mutation (n=1), p.T2031S (n=1) | 33 – 48 | 38 – 65 | STN bilat. | NA | 41.4 ± 12.4 M-, 8.2 ± 4.6 M+  (NC: 43.4 ± 17.0 M-) | 47.7 ± 13.1  M-S-, 17.8 ± 9.6  M-S+, 11.8 ± 4.5 M+S-, 6.2 ± 3.9 M+S+  (NC: 15.7 ± 9.0) | 50 ± 36  (NC: 64) | 9-10 (Long-term FU for two patients) | 10 | Favourable and comparable to patients without mutations but cognitive, behavioral and psychotic problems in the patient with p.T2031S mutation after 5 years. |
| Angeli et al. 2013 [6] | 5 | *LRRK2* | p.G2019S (n=4), p.G2019S + *GBA*-E326K (n=1) | 35 – 55 | NA^B^ | STN*** | NA | 65.4 ± 14.9 M-, 10.8 ± 5.1 M+  (NC: 47.6 ± 14.8 M-) | 69.2 ± 12.4  M-S-, 30.6 ± 16.1  M-S+  (24.6 ± 11.3 M-S+) | 53  (NC: 48) | 1 – 5 | 9 | Favourable and comparable to patients without mutations. No reported cognitive problems. |
| Gómez-Esteban et al. 2008 [7] | 4 | *LRRK2* | p.R1441G | 29 – 55 | 41 – 65 | STN bilat. | + | 48.5 ± 18.5 M-, 18.0 ± 7.4 M+  (NC: 42.5 ± 10.6 M-) | 39.7 ± 17.7  M-S+, 16.0 ±/–7.7 M+S+  (NC: 26.1 ± 8.4 M-S+) | 18  (NC: 39) | 0.5 | 10 | Poorer response compared to patients without mutation. |
| Johansen et al. 2011 [8] | 3 | *LRRK2* | p.G2019S | 43 – 57 | 50 – 69 | STN bilat. | + | NA for individual genes  (NC: 35.7 ± 6.7 M-) | NA for individual genes  (NC: 19.7 ± 5.5 M-S+) | NA  (NC: 44.8) | 5 | 9 | Favourable and comparable to patients without mutations. |
| Lesage et al. 2007 [9] | 3 | *LRRK2* | p.G2019S (n=2), p.T2031S (n=1) | 34 – 45 | 41 – 66 | STN*** | NA | 14 M+ (n=1),  NA (n=2) | 27 M-S+ (n=1),  17 M-S+ and  32 M-S- (n=1) | NA | 7 (Long-term FU for one patient) | 9 | Favourable to motor symptoms but depression and psychosis in the patient with p.T2031S mutation. |
| Hatano et al. 2014 [12] | 1 | *LRRK2* | p.R1441G and p.G2385R | 28 | 39 | STN bilat. | + | NA | NA | NA | 2 | 7 | Poor motor response with severe psychiatric problems at 1 year after operation. |
| Puschmann et al. 2012 [14] | 1 | *LRRK2* | p.N1437H (c.4309A>C) | 50 | 69 | STN bilat. | + | NA | 65 M-S+ | NA | 0.5 | 8 | Poor motor outcome. Patient had also severe depression and suicidality and she finally committed suicide 6.5 months after DBS implantation. |
| Perju-Dumprava et al. 2012 [15] | 1 | *LRRK2* | p.Y1699C | 43 | 48 | STN bilat. | NA | 54 M-,  32 M+ | 26 M-S+, 15 M+S+ | 52 M-,  53 M+ | 2.5 | 10 | Favourable outcome. No changes in neuropsychological test parameters 6 months postoperatively. |
| Breit et al. 2010 [16] | 1 | *LRRK2* | p.R793M | 42 | 60 | STN bilat. | NA | NA | NA | 64  (1year),  56  (8 year) | 8 | 8 | Favourable outcome. |
| Lohmann et al. 2008 [18] | 14 | *PRKN* | One mutation: ex6hetdupl, ex6hetdel, Arg256Cyshet [n=2], Ala398Thrhet, ex7hetdupl, and exhet3del; Hom. or compound het.: ex5hetdel– c.255delAhet, ex3hetdel–prom-ex1hetdel, ex2-4hetdupl– ex3hetdel, Cys289Glyhom, ex5hetdel–Cys441Arghet, ex2hetdel– ex3hetdel and ex4-7hetdel–IVS7-1GC | 14 – 52 | 32 – 67 | STN bilat. | NA | *One mutation:*  54.3 ± 13.9 M-,  11.6 ± 12.7 M+  *Two mutations:*  55.4 ± 17.3 M-,  14.5 ± 10 M+  (NC: 51.9 ± 18.3 M-) | *One mutation:*  38.4 ± 16.8  M-S-,  12.7 ± 11.2 M+S-,  17.8 ± 11.2  M-S+,  10.8 ± 10.1  M+S+  *Two mutations:*  47.7 ± 12.8  M-S-,  17 ± 10.9  M-S+,  14.5 ± 12.5  M-S+,  9.3 ± 8.6 M+S+  (NC: 17.9 ± 15.1 M-S+) | *One mutation:*  69 ± 15  *Two mutations:*  77 ± 14  (MC: 65.5) | 1 – 2 except 3 years for one patient with two PRKN mutations | 10 | Motor response was favourable and comparable to patients without mutations, but more cognitive problems in homozygous and compound heterozygous patients compared to patients without mutations. |
| Moro et al. 2008 [19] | 11 | *PRKN* | One mutation: delEx6, duplEx5, 867C>T, 1306G>C, delEx5-12; Hom. or compound het.: 202delA [n=2], delEx3-4, delEx3 + 1142-3delGA, delEx2-5 + duplEx8, delEx7-9 | 15 – 40 | 31 – 66 | STN bilat. | NA | 35 – 66 (MV = 49.5) | NA | *Short FU:*  36  *Long FU:*  42  (NC:  Short FU 56  Long FU 44) | 3 – 6 | 9 | Favourable and comparable to patients without mutations in long-term follow-up. |
| Angeli et al. 2013 [6] | 5 | *PRKN* | Hom.: c.101_102delAG, c.1289G>A p.G430D and c.823C>T, p.Arg275Trp, c.337_376del and c.465–466del, Hom. deletion of exon 3 and 4, c.823C>T; p.Arg275Trp and het. duplication of exon 6 | 7 – 36 | NA^B^ | GPi (n=3), STN*** (n=2) | NA | *All:*  57.0 ± 11.2 M-, 21.0 ± 6.4 M+  *GPi:*  53.3 ± 13.9 M-  *STN:*  62.5 ± 3.5 M-  (NC:  STN: 47.6 ± 14.8 M-GPi: 40.5 ± 13.4 M-) | *GPi:*  43.3 ± 16.4  M-S-  42.0 ± 19.0  M-S+  27.3 ± 17.6  M+S+  *STN:*  84.0 ± 22.6  M-S-,  43.0 ± 0.0 M-S+  23.5 ± 6.4 M+S+  (NC:  STN: 24.6 ± 11.3 M-S+ GPi: 51.0 ± 7.1 M-S+) | *GPi:*  21  *STN:*  31  (NC:  STN: 48  GPi: -28) | 1 – 5 | 9 | Good to motor symptoms without cognitive problems. The percentage improvement in the UPDRS III score was better with STN-DBS than with GPi-DBS. |
| Romito et al. 2005 [20] | 5 | *PRKN* | G828A and Dupl ex1, DelAG 202–203, C1101T, G535A, Dupl ex1 | 27 – 45 | 42 – 63 | STN bilat. | + | 57.3 ± 9.3 M-,  22.8 ± 7.3 M+  (NC: 59.7 ± 11.3 M-) | 25.2 ± 10.0 M-S+,  21.8 ± 7.5 M+S+  (NC: 29.0 ± 12.3 M-S+) | 56  (NC: 51.4) | 1 – 3 | 10 | Favourable and comparable to patients without mutations. |
| Johansen et al. 2011 [8] | 4 | *PRKN* | Het. c.delEx3, Het. p.R275W, Het. c.duplEx7, Hom. c.delEx5 (GPi) | 35 – 46 | 50 – 59 | STN bilat. (n=3), GPi unilat. (n=1) | + | NA for individual genes  (NC: 35.7 ± 6.7 M-) | NA for individual genes  (NC: 19.7 ± 5.5 M-S+) | NA  (NC: 44.8) | 5 – 7 | 9 | Favourable and comparable to patients without mutations. |
| Kim et al. 2014 [21] | 3 | *PRKN* | NA | 21.7 ± 8.5 | 49.7 ± 16.2 | STN bilat. | NA | 49.8 ± 24.5 M-,  18.3 ± 7.8 M+  (NC: 38.3 ± 10.6 M-) | 24.7 ± 14.0 M-S+,  22.2 ± 14.9 M+S+  (NC: 17.2 ± 5.5 M-S+) | 37.1± 45.4  (NC: 54.6 ± 13.9) | 2 – 5 | 10 | Favourable and comparable to patients without mutations. |
| Sayad et al. 2016 [2] | 2 | *PRKN* | Het. c. 458C>G , | 48 | NA | STN bilat. | + | 46 M-,  28 M+ | 51 M-S+, 30 M+S+ | -10.1 | 2 | 10 | Poor response. |
|  |  |  | Het. c. 1204C>T | 48 |  |  | + | 49 M-,  32 M+  (NC: 51.7 ± 14.4 M-) | 51 M-S+, 47 M+S+  (NC: 38.5 ± 16.6 M-S+) | -4.1  (NC: 25.5) |  |  |  |
| Thompson et al. 2013 [23] | 2 | *PRKN* | Hom., specific mutation NA | 26 (Gpi), 30 (STN) | NA | STN bilat. (n=1), GPi bilat. (n=1) | NA | *GPi:*  57 M-,  50 M+  *STN:*  47 M-,  21 M+ | NA | NA | 3 (STN), 8 (GPi) | 6 | Favourable outcome. |
| Nakahara et al. 2014 [26] | 1 | *PRKN + PINK1* | Hom. parkin mutation (p.T175PfsX2) + het. PINK1 mutation (p.R58-V59insGR) | 15 | 60 | STN bilat. | + | 86 M-,  25 M+ | 33 M-S+,  21 M+S+ | 62 | 0.7 | 9 | Favourable outcome. |
| Lefaucheur et al. 2010 [27] | 1 | *PRKN* | Compound het. mutations of the *PRKN* gene, [c.101_102delAG (p.Gln34ArgfsX5) + c.155delA (p.Asn52MetfsX29)] | 25 | 69 | STN*** | NA | NA | NA | 55 | 0.5 | 8 | Favourable to motor symptoms without cognitive problems. |
| Wickremaratchi et al. 2009 [28] | 1 | *PRKN* | Compund het. exon 2/exon 2 1 3 deletion in the *PRKN* | 8 | 46 | Zona incerta bilat. | NA | 68 M-,  22 M+ | NA M-S+  24 M+S+ | NA M-,  64.7 M+ | 0.5 | 9 | Favourable outcome. |
| Capecci et al. 2004 [30] | 1 | *PRKN* | Hom. deletion in exon 3 | 22 | NA | STN bilat. | + | 45 M-,  5 M+ | 7 M-S+,  3 M+S+ | 84.4 | 1 | 8 | Favourable outcome. |
| Angeli et al. 2013 [6] | 16 | *GBA* | R463C/R463C, L444P/E326K, N370S, D409H, recNcil, R463C, N188S, R275Q, IVS2 + 1 G>A, L444P, E326K/E326K, E326K (n=3), E326K and *LRRK2* p.G2019S, T369M and *PRKN* c.1310C>T | 34 – 58 | NA^B^ | STN*** (n=13), GPi (n=2), VIM (n=1) | NA | *All:*  51.3 ± 14.0 M-,  18.0 ± 15.4M+  *GPi:*  64.5 ± 21.9M-  *STN:*  50.5 ± 12.4 M-  *VIM:*  35 M-  (NC:  STN: 47.6 ± 14.8 M-GPi: 40.5 ± 13.4 M-) | *GPi:*  66.5 ± 19.1 M-S-,  50.0 ± 19.8  M-S+, 41.0 ± 15.6 M+S+  *STN:*  56.1 ± 18.8 M-S-,  28 ± 11.4 M-S+,  15.9 ± 10.4 M+S+  *VIM:*  35 M-S-,  20 M-S+,  8 M+S+  (NC:  STN: 24.6 ± 11.3 M-S+ GPi: 51.0 ± 7.1 M-S+) | *GPi:*  22  *STN:*  40  *VIM:*  43  (NC:  STN: 48  GPi: -28) | 1 – 5 | 9 | Favourable motor response but faster rate of cognitive decline compared to patients without mutations. The percentage improvement in the UPDRS III score “OFF- medication” was better with bilateral STN-DBS and VIM-DBS than with GPi-DBS. |
| Weiss et al. 2012 [33] | 3 | *GBA* | p.N370S (n=1) and p.L444P (n=2) | 47 – 54 | 65 – 69 | STN*** | NA | 26 and 53 M-,  14 and 19 M+,  NA (n=1)  (NC: 31 – 63 M-) | 56­ – 71  M-S-,  21 – 45  M-S+,  32 – 48 M+S-,  20–45  M+S+  (NC: 21 ­– 42 M-S+) | 30 – 75  (NC: 22 – 54) | 6 – 10 | 11 | Favourable outcome but substantial increase of axial motor impairment in the long-term with declining therapeutic response in *GBA* carriers. *GBA* carriers developed also a significant cognitive impairment. |
| Lesage et al. 2011 [34] | 2 | *GBA* | Hom. p.N370S | 52 | NA | STN bilat. | NA | NA | NA | NA | NA | 5 | Favourable outcome. |
|  |  |  | c.1263del+RecTL | 21 | 24 |  |  |  |  |  | 2 |  | Some clinical benefit 2 years after DBS but problems with postural instability. |
| Martikainen et al. 2015 [35] | 1 | *SNCA* | Het. c.158C>A (p.A53E) | 42 | 46 | STN bilat. | NA | 31 M-,  8 M+ | NA | NA | 3.5 | 9 | Favourable motor outcome in the short-term but poor in the long-term follow-up.  Response for motor fluctuations remained satisfactory but the cognitive and mental state of the patient deteriorated to a state of practical immobility. |
| Shimo et al. 2014 [37] | 1 | *SNCA* | *SNCA* duplication | 35 | 41 | STN bilat. | + | 27 M-,  10 M+ | 13 M-S+ | 51.9 | 4 | 9 | Favourable motor outcome without cognitive or psychiatric problems. |
| Antonini et al. 2012 [38] | 1 | *SNCA* | *SNCA* duplication at 4q22.1 | 41 | 46 | STN bilat. | + | 28 M-,  10 M+ | 16 M-S+, 10 M+S+ | 42.9 | 2 | 9 | Favourable outcome in short-term follow-up but patient developed visual hallucinations and cognitive deterioration and died two years after operation due to metastatic breast cancer. |
| Fleury et al. 2013 [40] | 2 | *VPS35* | p.D620N | 49 | 60 | STN bilat. | NA | 58 M-,  17 M+ | 32 M-S-, 18 M-S+, 18 M+S-, 15 M+S+ | 76  (1 year)  69  (8 years) | 8 | 8 | Favourable outcome. |
|  |  |  |  | 45 | 55 |  |  | 28 M-,  15 M+ | NA | 36  (1 year) | 1 |  | Tremor, akinesia and rigidity improved markedly but patient’s walking difficulties worsened with an increased frequency of freezing episodes and falls after surgery (problems disappeared after levodopa intake with the STN-DBS switched on). |
| Chen et al. 2017 [41] | 1 | *VPS35* | p.D620N | 42 | 55 | STN bilat. | + | 42 M-,  15 M+ | 35 M-S-, 22 M-S+, 15 M+S-, 13 M+S+ | 37 | 5 | 9 | Favourable outcome. |
| Borellini et a. 2017 [44] | 1 | *PINK1* | Hom. L347P | 30 | 49 | GPi | NA | 44 M- | 32 M+S+ | 27 | 0.1 | 7 | Moderate outcome. |
| Nakahara et al. 2014 [26] | 1 | *PRKN + PINK1* | Hom. parkin mutation (p.T175PfsX2) + het. PINK1 mutation (p.R58-V59insGR) | 15 | 60 | STN bilat. | + | 86 M-,  25 M+ | 33 M-S+,  21 M+S+ | 62 | 0.7 | 9 | Favourable outcome. |
| Johansen et al. 2011 [8] | 1 | *PINK1* | Het. p.G411S | 50 | 59 | STN bilat. | + | NA for individual genes  (NC: 35.7 ± 6.7 M-) | NA for individual genes  (NC: 19.7 ± 5.5 M-S+) | NA  (NC: 44.8) | 5 | 9 | Favourable and comparable to patients without mutations. |
| Moro et al. 2008 [19] | 1 | *PINK1* | Hom. c.509T>G (p.V170G) | 31 | 61 | STN bilat. | NA | 35.5 M- | NA | *Short FU*:  46.5  *Long FU*:  43.7  (NC:  Short FU 56  Long FU 44) | 3 – 6 | 9 | Favourable and comparable to patients without mutations. |

^AAO = Age at disease onset (years), AAD = Age at DBS operation (years), LP = Specific lead position (reported or not), % = The percentage improvement of the UPDRS-III score after DBS** , FU = Follow-up after surgery (years), NA = Not available, M-/+ = Medication OFF/ON, S-/+ = Stimulation OFF/ON, MV = Mean value, NC = Mutation non-carriers, A = The mean time from PD onset to surgery was 11.4 years (SD 6.2), B = Mean duration of PD (years) at DBS assessment: PRKN = 25.2 ± 12.8, GBA = 11.2 ± 5.0, LRRK2 = 12.1 ± 1.8.^

^* Parameters are reported in the table as in the original articles.^

^** If the percentage improvement was not reported directly in the original article but UPDRS-III scores were available, we calculated the percentage improvement from the change of UPDRS-III score in the preoperative M- condition compared to the postoperative M-S+ condition (((Pre-op. UPDRS-III M-) ­– (Post.op. UPDRS-III M-S+)) / (Pre-op. UPDRS-III M-) x 100)­.^

^*** The study did not specify whether the implantation was uni- or bilateral.^

| *Gene* | *Studies (n)* | *Patients (n)* | *Target* | *Outcome* |
| --- | --- | --- | --- | --- |
| *LRRK2* | 11 | 56* | STN: n=56 (100.0 %) | Mostly favourable motor outcome. Three studies with six patients (10.7 %) reported poor motor outcomes. Both patients with the *LRRK2* p.T2031S (c.6091A>T) mutation (n = 2) developed neuropsychiatric problems 5-7 years after implantation. The outcome appears poor in patients with *LRRK2* p.R1441G (c.4321C>G) mutations (n = 5) whereas it appears excellent in patients with *LRRK2* p.G2019S (c.6055G>A) mutations. |
| *PRKN* | 12 | 50** | STN: n=44 (88.0 %)  GPi: n=5 (10.0 %)  Zona incerta: n=1 (2.0 %) | Forty-seven patients (94.0 %) had favourable long-term motor outcomes. One patient (2.0 %) was reported to have modest outcome and one study with two patients (4.0 %) reported poor benefit. |
| *GBA* | 3 | 21*** | STN: n=18 (85.7 %)  GPi: n=2 (9.5 %)  VIM: n=1 (4.8 %) | Eighteen patients were reported to have favourable and three patients modest long-term motor outcomes. One study reported better outcomes with STN-DBS and VIM-DBS than with GPi-DBS. *GBA* mutation carriers developed cognitive impairment faster than patients without mutations. |
| *SNCA* | 3 | 3 | STN: n=4 (100.0 %) | Favourable motor outcome but two of three patients developed cognitive or neuropsychiatric problems a few years after implantation. |
| *VPS35* | 2 | 3 | STN: n=3 (100.0 %) | Favourable motor outcome. |
| *PINK1* | 4 | 4** | STN: n=3 (75.0 %)  GPi: n=1 (25.0 %) | Favourable motor outcome in three cases and moderate in one case. |

^STN = Subthalamic nucleus, GPi = Globus pallidus interna, VIM = Ventral intermediate nucleus, NA = not available.^

^* One patient had also PRKN mutation and one had GBA mutation.^

^**One patient had both PRKN and PINK1 mutations.^

^*** Two studies reported partially same patients, but it was not possible to separate individual patients that were reported twice. One patient had also LRRK2 mutation and one had PRKN mutation.^

**References**

[1]. Healy DG, Falchi M, O'Sullivan SS*, et al.* Phenotype, genotype, and worldwide genetic penetrance of LRRK2-associated Parkinson's disease: a case-control study. *Lancet Neurol*. 2008 **7:** 583-590.

[2]. Sayad M, Zouambia M, Chaouch M*, et al.* Greater improvement in LRRK2 G2019S patients undergoing Subthalamic Nucleus Deep Brain Stimulation compared to non-mutation carriers. *BMC Neurosci*. 2016 **17:** 6.

[3]. Greenbaum L, Israeli-Korn SD, Cohen OS*, et al.* The LRRK2 G2019S mutation status does not affect the outcome of subthalamic stimulation in patients with Parkinson's disease. *Parkinsonism Relat Disord*. 2013 **19:** 1053-1056.

[4]. Schüpbach M, Lohmann E, Anheim M*, et al.* Subthalamic nucleus stimulation is efficacious in patients with Parkinsonism and LRRK2 mutations. *Mov Disord*. 2007 **22:** 119-122.

[5]. Pal GD, Hall D, Ouyang B*, et al.* Genetic and Clinical Predictors of Deep Brain Stimulation in Young-Onset Parkinson's Disease. *Mov Disord Clin Pract*. 2016 **3:** 465-471.

[6]. Angeli A, Mencacci NE, Duran R*, et al.* Genotype and phenotype in Parkinson's disease: lessons in heterogeneity from deep brain stimulation. *Mov Disord*. 2013 **28:** 1370-1375.

[7]. Gómez-Esteban JC, Lezcano E, Zarranz JJ*, et al.* Outcome of bilateral deep brain subthalamic stimulation in patients carrying the R1441G mutation in the LRRK2 dardarin gene. *Neurosurgery*. 2008 **62:** 857-862; discussion 862-853.

[8]. Johansen KK, Jørgensen JV, White LR, Farrer MJ, Aasly JO. Parkinson-related genetics in patients treated with deep brain stimulation. *Acta Neurol Scand*. 2011 **123:** 201-206.

[9]. Lesage S, Janin S, Lohmann E*, et al.* LRRK2 exon 41 mutations in sporadic Parkinson disease in Europeans. *Arch Neurol*. 2007 **64:** 425-430.

[10]. Gaig C, Ezquerra M, Marti MJ, Muñoz E, Valldeoriola F, Tolosa E. LRRK2 mutations in Spanish patients with Parkinson disease: frequency, clinical features, and incomplete penetrance. *Arch Neurol*. 2006 **63:** 377-382.

[11]. Goldwurm S, Di Fonzo A, Simons EJ*, et al.* The G6055A (G2019S) mutation in LRRK2 is frequent in both early and late onset Parkinson's disease and originates from a common ancestor. *J Med Genet*. 2005 **42:** e65.

[12]. Hatano T, Funayama M, Kubo SI*, et al.* Identification of a Japanese family with LRRK2 p.R1441G-related Parkinson's disease. *Neurobiol Aging*. 2014 **35:** 2656.e2617-2656.e2623.

[13]. Stefani A, Marzetti F, Pierantozzi M*, et al.* Successful subthalamic stimulation, but levodopa-induced dystonia, in a genetic Parkinson's disease. *Neurol Sci*. 2013 **34:** 383-386.

[14]. Puschmann A, Englund E, Ross OA*, et al.* First neuropathological description of a patient with Parkinson's disease and LRRK2 p.N1437H mutation. *Parkinsonism Relat Disord*. 2012 **18:** 332-338.

[15]. Perju-Dumbrava LD, McDonald M, Kneebone AC, Long R, Thyagarajan D. Sustained response to deep brain stimulation in LRRK2 parkinsonism with the Y1699C mutation. *J Parkinsons Dis*. 2012 **2:** 269-271.

[16]. Breit S, Wächter T, Schmid-Bielenberg D*, et al.* Effective long-term subthalamic stimulation in PARK8 positive Parkinson's disease. *J Neurol*. 2010 **257:** 1205-1207.

[17]. Aasly JO, Vilariño-Güell C, Dachsel JC*, et al.* Novel pathogenic LRRK2 p.Asn1437His substitution in familial Parkinson's disease. *Mov Disord*. 2010 **25:** 2156-2163.

[18]. Lohmann E, Welter ML, Fraix V*, et al.* Are parkin patients particularly suited for deep-brain stimulation? *Mov Disord*. 2008 **23:** 740-743.

[19]. Moro E, Volkmann J, König IR*, et al.* Bilateral subthalamic stimulation in Parkin and PINK1 parkinsonism. *Neurology*. 2008 **70:** 1186-1191.

[20]. Romito LM, Contarino MF, Ghezzi D, Franzini A, Garavaglia B, Albanese A. High frequency stimulation of the subthalamic nucleus is efficacious in Parkin disease. *J Neurol*. 2005 **252:** 208-211.

[21]. Kim HJ, Yun JY, Kim YE*, et al.* Parkin mutation and deep brain stimulation outcome. *J Clin Neurosci*. 2014 **21:** 107-110.

[22]. Hassin-Baer S, Hattori N, Cohen OS, Massarwa M, Israeli-Korn SD, Inzelberg R. Phenotype of the 202 adenine deletion in the parkin gene: 40 years of follow-up. *Mov Disord*. 2011 **26:** 719-722.

[23]. Thompson AJ, Scholz SW, Singleton AB, Hardwick A, McFarland NR, Okun MS. Variability in clinical phenotypes of heterozygous and homozygous cases of Parkin-related Parkinson's disease. *Int J Neurosci*. 2013 **123:** 847-849.

[24]. Genç G, Apaydın H, Gündüz A*, et al.* Successful treatment of Juvenile parkinsonism with bilateral subthalamic deep brain stimulation in a 14-year-old patient with parkin gene mutation. *Parkinsonism Relat Disord*. 2016 **24:** 137-138.

[25]. Moll CK, Buhmann C, Gulberti A*, et al.* Synchronized cortico-subthalamic beta oscillations in Parkin-associated Parkinson's disease. *Clin Neurophysiol*. 2015 **126:** 2241-2243.

[26]. Nakahara K, Ueda M, Yamada K*, et al.* Juvenile-onset parkinsonism with digenic parkin and PINK1 mutations treated with subthalamic nucleus stimulation at 45 years after disease onset. *J Neurol Sci*. 2014 **345:** 276-277.

[27]. Lefaucheur R, Derrey S, Guyant-Maréchal L, Chastan N, Maltête D. Whatever the disease duration, stimulation of the subthalamic nucleus improves Parkin disease. *Parkinsonism Relat Disord*. 2010 **16:** 482-483.

[28]. Wickremaratchi MM, Majounie E, Morris HR*, et al.* Parkin-related disease clinically diagnosed as a pallido-pyramidal syndrome. *Mov Disord*. 2009 **24:** 138-140.

[29]. Lesage S, Magali P, Lohmann E*, et al.* Deletion of the parkin and PACRG gene promoter in early-onset parkinsonism. *Hum Mutat*. 2007 **28:** 27-32.

[30]. Capecci M, Passamonti L, Annesi F*, et al.* Chronic bilateral subthalamic deep brain stimulation in a patient with homozygous deletion in the parkin gene. *Mov Disord*. 2004 **19:** 1450-1452.

[31]. Khan NL, Graham E, Critchley P*, et al.* Parkin disease: a phenotypic study of a large case series. *Brain*. 2003 **126:** 1279-1292.

[32]. Lythe V, Athauda D, Foley J*, et al.* GBA-Associated Parkinson's Disease: Progression in a Deep Brain Stimulation Cohort. *J Parkinsons Dis*. 2017 **7:** 635-644.

[33]. Weiss D, Brockmann K, Srulijes K*, et al.* Long-term follow-up of subthalamic nucleus stimulation in glucocerebrosidase-associated Parkinson's disease. *J Neurol*. 2012 **259:** 1970-1972.

[34]. Lesage S, Anheim M, Condroyer C*, et al.* Large-scale screening of the Gaucher's disease-related glucocerebrosidase gene in Europeans with Parkinson's disease. *Hum Mol Genet*. 2011 **20:** 202-210.

[35]. Martikainen MH, Päivärinta M, Hietala M, Kaasinen V. Clinical and imaging findings in Parkinson disease associated with the A53E SNCA mutation. *Neurol Genet*. 2015 **1:** e27.

[36]. Perandones C, Aráoz Olivos N, Raina GB*, et al.* Successful GPi stimulation in genetic Parkinson's disease caused by mosaicism of alpha-synuclein gene duplication: first description. *J Neurol*. 2015 **262:** 222-223.

[37]. Shimo Y, Natori S, Oyama G*, et al.* Subthalamic deep brain stimulation for a Parkinson's disease patient with duplication of SNCA. *Neuromodulation*. 2014 **17:** 102-103.

[38]. Antonini A, Pilleri M, Padoan A*, et al.* Successful subthalamic stimulation in genetic Parkinson's disease caused by duplication of the α-synuclein gene. *J Neurol*. 2012 **259:** 165-167.

[39]. Ahn TB, Kim SY, Kim JY*, et al.* alpha-Synuclein gene duplication is present in sporadic Parkinson disease. *Neurology*. 2008 **70:** 43-49.

[40]. Fleury V, Wider C, Horvath J*, et al.* Successful long-term bilateral subthalamic nucleus deep brain stimulation in VPS35 Parkinson's disease. *Parkinsonism Relat Disord*. 2013 **19:** 707-708.

[41]. Chen YF, Chang YY, Lan MY, Chen PL, Lin CH. Identification of VPS35 p.D620N mutation-related Parkinson's disease in a Taiwanese family with successful bilateral subthalamic nucleus deep brain stimulation: a case report and literature review. *BMC Neurol*. 2017 **17:** 191.

[42]. Kumar KR, Weissbach A, Heldmann M*, et al.* Frequency of the D620N mutation in VPS35 in Parkinson disease. *Arch Neurol*. 2012 **69:** 1360-1364.

[43]. Sheerin UM, Charlesworth G, Bras J*, et al.* Screening for VPS35 mutations in Parkinson's disease. *Neurobiol Aging*. 2012 **33:** 838.e831-835.

[44]. Borellini L, Cogiamanian F, Carrabba G*, et al.* Globus pallidus internus deep brain stimulation in PINK-1 related Parkinson's disease: A case report. *Parkinsonism Relat Disord*. 2017 **38:** 93-94.

[45]. Valente EM, Salvi S, Ialongo T*, et al.* PINK1 mutations are associated with sporadic early-onset parkinsonism. *Ann Neurol*. 2004 **56:** 336-341.

[46]. Dufournet B, Nguyen K, Charles P*, et al.* Parkinson's disease associated with 22q11.2 deletion: Clinical characteristics and response to treatment. *Rev Neurol (Paris)*. 2017 **173:** 406-410.
